# EpCAM Expression in Lymph Node and Bone Metastases of Prostate Carcinoma: A Pilot Study

**DOI:** 10.3390/ijms17101650

**Published:** 2016-09-29

**Authors:** Anna K. Campos, Hilde D. Hoving, Stefano Rosati, Geert J. L. H. van Leenders, Igle J. de Jong

**Affiliations:** 1 Laboratory of Neuroimmunology, National Institute of Neurology and Neurosurgery “Manuel Velasco Suárez”, Avenida Insurgentes Sur 3877, La Fama, Tlalpan, 14269 Mexico City, Mexico; camposakaren@gmail.com; 2 Department of Urology, University Medical Center Groningen, University of Groningen, P.O. Box 30.001, Groningen 9700 RB, The Netherlands; i.j.de.jong@umcg.nl; 3 Department of Pathology, University Medical Center Groningen, University of Groningen, P.O. Box 30.001, Groningen 9700 RB, The Netherlands; s.rosati@umcg.nl; 4 Department of Pathology, Josephine Nefkens Institute, Erasmus MC, P.O. Box 2040, Rotterdam 3000 CA, The Netherlands; g.vanleenders@erasmusmc.nl

**Keywords:** molecular imaging, imaging target, prostate carcinoma, metastases, epithelial cell adhesion molecule (EpCAM), immunohistochemistry

## Abstract

There is an urgent need for new imaging modalities in prostate carcinoma staging. A non-invasive modality that can assess lymph node and bone metastases simultaneously is preferred. Epithelial cell adhesion molecule (EpCAM) is a membranous protein of interest as an imaging target since it is overexpressed in prostatic carcinoma compared with benign prostate epithelium and compared with stroma. However, EpCAM expression in lymph node metastases is sparsely available in the literature and EpCAM expression in bone metastases is yet unknown. The current study evaluates the expression of EpCAM in prostate carcinoma lymph nodes, in matched normal lymph nodes, in prostate carcinoma bone metastases, and in normal bone by immunohistochemistry. EpCAM was expressed in 100% of lymph node metastases (21 out of 21), in 0% of normal lymph nodes (0 out of 21), in 95% of bone metastases (19 out of 20), and in 0% of normal bone (0 out of 14). Based on these results, EpCAM may be a feasible imaging target in prostate carcinoma lymph node and bone metastases. Prospective clinical trials are needed to confirm current results. Preoperative visualization of prostate carcinoma metastases will improve disease staging and will prevent unnecessary invasive surgery.

## 1. Introduction

Epithelial cell adhesion molecule (EpCAM), also known as CD326 and 17-1A antigen, is a transmembrane glycoprotein originally identified as a marker for carcinoma [1]. EpCAM functions as a cell adhesion molecule in benign and malignant epithelial cells [2]. However, its role also includes signaling, cell migration, proliferation, and differentiation [1,3,4].

EpCAM has been found expressed in various types of carcinoma, including colon and rectum, gallbladder, liver, esophagus, lung, head and neck, pancreas, ovarian, breast, and prostate carcinoma [3,4,5].

There are several studies that show a significantly elevated expression of EpCAM in prostatic carcinoma compared with benign prostate epithelium [5,6,7]. Next, Poczatek et al. and Benko et al. found that there was no expression of EpCAM in prostate stroma [5,7]. Based on these results, EpCAM would be an attractive specific target for imaging purposes in prostate carcinoma. Several preclinical trials targeted EpCAM for fluorescent and nuclear imaging [8,9,10,11]. Results of preclinical fluorescent imaging are promising for the accurate assessment of tumor margins intraoperatively [11,12]. Thus, nuclear imaging of EpCAM would be of special interest for prostate carcinoma staging and for monitoring of treatment response. The importance of correct staging lies in the fact that staging has huge treatment implications. Metastasized prostate carcinoma cannot be treated curatively. However, current imaging modalities in prostate carcinoma staging have limitations.

Prostate carcinoma lymph node staging is limited by poor sensitivity and specificity of anatomical imaging modalities [13,14,15]. Molecular imaging of lymph node metastases by positron emission tomography (PET) is booming nowadays. ^11^C- or ^18^F-choline PET/CT has good specificity for the detection of lymph node metastases. However, the sensitivity ranges from 10% to 73% [16,17]. This might result from the target choline, which is not prostate carcinoma-specific. Pelvic lymph node dissection remains the gold standard for nodal staging. Lymph node dissection has been associated with regional disease control and better outcome, but this can lead to poor patient outcome (complications such as lymph edema and thrombosis) and the overtreatment of patients with a low risk of metastases [18,19,20]. Therefore, a noninvasive imaging modality to assess lymph node metastases would be preferred.

For the assessment of bone metastases, skeletal scintigraphy is the most sensitive method. However, false positive skeletal scintigraphy occurs, for example, from degenerative disease, inflammation, and trauma. Furthermore, skeletal scintigraphy is hampered by the osteoblastic response that accompanies bone healing, which can also lead to a false positive diagnosis of disease progression [21,22,23]. Furthermore, skeletal scintigraphy lacks anatomical detail, and treatment response can take about 6 to 8 months before response can be visualized [24,25].

To summarize, there is an urgent need for new imaging modalities in prostate carcinoma staging. A non-invasive modality, that can assess lymph node and bone metastases simultaneously, is preferred. This imaging modality should be based on a prostate carcinoma-specific target such as EpCAM.

However, EpCAM expression in prostate carcinoma metastases has been evaluated in only one study. EpCAM was significantly overexpressed in metastasized prostate carcinoma compared with benign prostate hyperplasia, which served as a normal control [6]. Since specimens were collected at autopsies, results should be interpreted with caution. A decay of antigens can be seen in postmortem specimens.

The current research evaluates the expression of EpCAM in prostate carcinoma lymph nodes, in matched normal lymph nodes, in prostate carcinoma bone metastases, and in normal bone in order to determine the feasibility of EpCAM as a specific marker for prostate carcinoma staging.

EpCAM was expressed in 100% of lymph node metastases (21 out of 21), in 0% of normal lymph nodes (0 out of 21), in 95% of bone metastases (19 out of 20), and in 0% of normal bone (0 out of 14). Based on these results, EpCAM may be a feasible imaging target in prostate carcinoma lymph node and bone metastases. Preoperative visualization of these metastases will improve disease staging and will prevent unnecessary invasive surgery.

## 2. Results

After immunohistochemistry, one prostate carcinoma lymph node metastasis and four prostate carcinoma bone metastases were not evaluable due to bad morphology. The matched normal lymph node was also excluded from statistical analysis. In the end, 20 bone metastases (of 20 patients), 14 normal bone (of 14 patients) and 21 lymph node metastases, and 21 normal lymph node metastases (of 16 patients) were available for statistical analysis. Clinicopathological parameters of these patients are presented in Table 1, Table 2 and Table 3.

EpCAM expression was observed in 19 out of 20 bone metastases (95%) and was absent in 14 out of 14 cases of normal bone (100%). Median EpCAM expression in bone metastases (TIS) was 12. After either hormonal therapy or radiotherapy, TIS was high. Even after both hormonal therapy and radiotherapy, EpCAM expression was high (Table 2).

**Figure 1:**
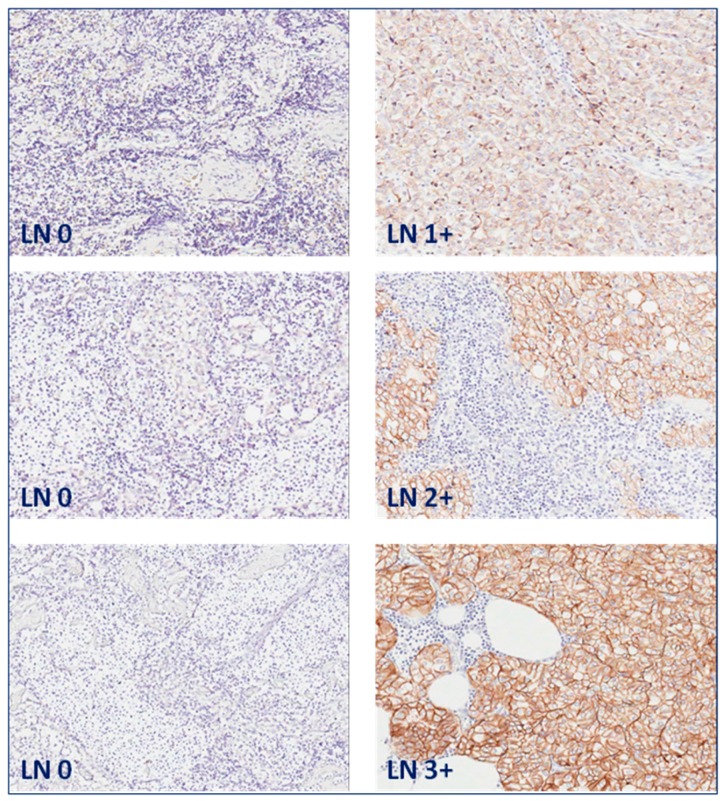
EpCAM expression in lymph node (LN) metastases. Matched normal lymph nodes without EpCAM expression (**left** panel) and lymph node metastases of prostate carcinoma with EpCAM expression of intensity score 1: weak; 2: moderate; and 3: strong (**right** panel). Original magnification: 200×.

**Figure 2:**
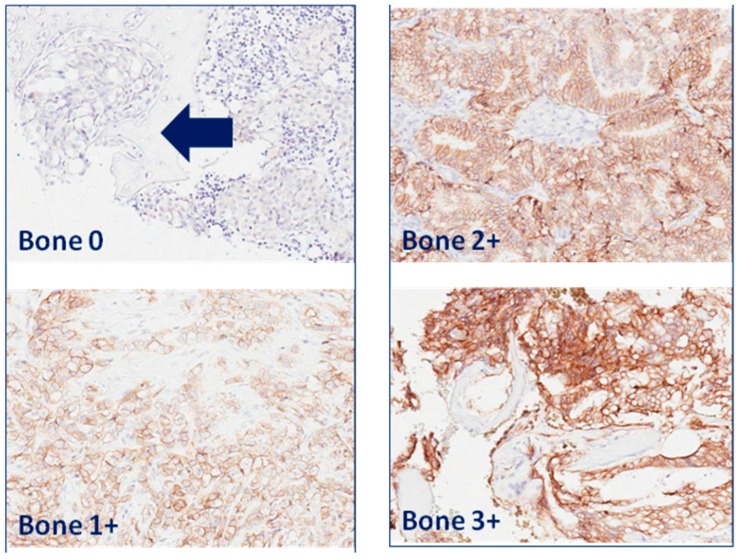
EpCAM expression in prostate carcinoma bone metastases. Bone metastases with EpCAM expression of intensity score 1: weak; 2: moderate; and 3: strong. Arrow points towards an area of bone metastasis in bone without EpCAM expression. Original magnification: 200×.

EpCAM expression was membranous in prostate carcinoma cells of lymph node (Figure 1) and bone metastases (Figure 2).

## 3. Discussion

In the current study, EpCAM was expressed in 100% of prostate carcinoma lymph node metastases and 95% of prostate carcinoma bone metastases. The median EpCAM expression was high, with a TIS of 8 and 12 for lymph node and bone metastases, respectively. EpCAM was not expressed in matched normal lymph nodes and in non-matched normal bone.

EpCAM expression in prostate carcinoma metastases has been evaluated in only one study previously. However, bone metastases were not included. EpCAM was significantly overexpressed in metastasized prostate carcinoma compared with benign prostate hyperplasia, which served as a normal control [6]. Since specimens were collected at autopsies, results should be interpreted with caution.

The current study is the first to compare EpCAM expression between matched normal lymph nodes and lymph node metastases and to evaluate EpCAM expression in bone metastases and normal bone. Absent EpCAM expression in normal lymph nodes and normal bone supports the use of EpCAM as a target with high specificity for prostate carcinoma.

Went et al. and Benko et al. investigated a possible correlation between EpCAM expression and nodal stage (N0, N1, or N2) in primary prostate carcinoma. EpCAM expression was defined as negative, weak to moderate and strong [1]. No correlation was found, presumably caused by small sample sizes of 10 and 3 primary prostate carcinoma cases, respectively. Moreover, lymph node specimens were not available in both studies [1,7].

In previous literature EpCAM overexpression was described as TIS > 4 [26]. In the current study, EpCAM overexpression was found in both lymph node and bone metastases regardless of previous treatment. This is in agreement with a study from Benko et al. in which a correlation was found between high EpCAM expression and biochemical recurrence of prostate carcinoma [7]. Next, EpCAM expression was seen in 82.3% of salvage prostatectomy specimens taken from patients with locally recurrent prostate carcinoma after external beam radiotherapy or brachytherapy [27].

## 4. Materials and Methods

Formalin-fixated paraffin-embedded prostate carcinoma lymph node metastases, normal lymph nodes, prostate carcinoma bone metastases, samples of normal bone, and samples of normal colon were retrieved from the archives of the Department of Pathology of University Medical Center Groningen. Lymph node metastases (*n* = 22) and matched normal lymph nodes (*n* = 22) were available in 17 patients who underwent a pelvic lymph node dissection because of a suspicion of nodal involvement on computed tomography (CT). Of 12 patients, 1 prostate carcinoma lymph node metastasis and 1 normal lymph node was included; of 3 patients, 2 prostate carcinoma lymph node metastases and 2 normal lymph nodes were included; of 1 patient, 3 prostate carcinoma lymph node metastases and 3 normal lymph nodes were included.

Prostate carcinoma bone metastases (*n* = 24) were available in 24 patients who underwent surgery to confirm clinical suspicion of prostate carcinoma or to treat skeletal related events. Bone metastases were taken from several surgical procedures (biopsy (*n* = 6), osteosynthesis (*n* = 1), laminectomy (*n* = 6), extramedullary excision (*n* = 1), corporectomy (*n* = 2), during procedures of hip prosthesis implantation (*n* = 1), and other, unknown procedures (*n* = 7)). Matched normal bone was not available. Fourteen cases of non-matched normal bone were available in 14 patients who underwent hip prosthesis implantation. All tissue specimens were anonymously coded. According to Dutch law, no further Institutional Review Board approval was required (http://www.federa.org/).

Immunohistochemistry was performed in order to determine EpCAM expression. Normal colon was used as a positive control and omission of the primary antibody on positive control specimens served as a negative control. After deparaffinization with decreasing grades of alcohol, antigen retrieval was performed by incubation with 0.1% protease for 30 min at room temperature. Endogeneous peroxidase was blocked with 0.3% hydrogen peroxide in phosphate buffered saline (PBS) for 20 min. Slides were incubated with primary mouse monoclonal antibody anti-EpCAM (clone VU-1D9, Leica Biosystems, Newcastle, UK) diluted at 1:100 in 1% bovine serum albumin and phosphate buffered saline (BSA/PBS) for 1 h at room temperature. In the secondary step, slides were incubated with a rabbit anti-mouse antibody conjugated to polymer-horseradish peroxidase (DAKO, Glostrup, Denmark), diluted at 1:100 in 1% BSA/PBS with 1% AB serum. In the tertiary step, a goat anti-rabbit antibody conjugated to polymer-horseradish peroxidase (DAKO, Glostrup, Denmark) was used, diluted at 1:100 in 1% BSA/PBS with 1% AB serum. Both secondary and tertiary steps required incubation for 30 min at room temperature. Next, slides were immersed for 10 min in a solution of 0.05% 3,3’-diaminobenzidine (Sigma-Aldrich, Steinheim, Germany) and 0.03% hydrogen peroxide in PBS for visualization of the signal as brown staining. After washing with demineralized water, slides were slightly counterstained with hematoxylin, dehydrated, and mounted with Tissue Tec film (Sakura Finetek, Leiden, The Netherlands).

A pathologist (GJLHvL), blinded to clinical data, scored EpCAM immunoreactivity according to a previous established method [28]. This method determines a total immunostaining score (TIS), which is the product of a proportion score (PS) and an intensity score (IS). The PS represents the estimated amount of positively stained cells (0: none; 1: <10%; 2: 10%–50%; 3: 51%–80%; 4: >80%). IS describes the estimated staining intensity (0: no staining; 1: weak; 2: moderate; 3: strong). TIS (TIS = PS × IS) ranges from 0 to 12 with only 9 possible values (0, 1, 2, 3, 4, 6, 8, 9, or 12) [28].

Sensitivity and specificity of EpCAM expression were determined. Specificity of EpCAM expression could be determined for lymph node specimens only. For bone specimens, specificity could not be determined due to the lack of negative control bone specimens. Sensitivity is the percentage of cases with EpCAM expression out of the total amount of either histopathological proven lymph node metastases or histopathological proven bone metastases. Sensitivity is the percentage of cases without EpCAM expression in histopathological proven lymph nodes without prostate carcinoma metastasis. 

Descriptive statistics were used to describe the results. For ordinal data, the median is presented.

## 5. Conclusions

In conclusion, the purpose of the current study was to establish the feasibility of EpCAM as an imaging target for prostate carcinoma lymph node and bone metastases. EpCAM has proved to be a target with high sensitivity (100%) and specificity (100%) for lymph node metastases and high sensitivity (95%) and specificity (100%) for bone metastases. Based on previous literature, EpCAM may be used as imaging target for locally recurrent prostate carcinoma [7,27]. Based on the current study, EpCAM may be additionally used as an imaging target for prostate carcinoma lymph node and bone metastases. Preoperative visualization of metastases will improve disease staging and will prevent unnecessary invasive surgery. Prospective clinical trials are needed to confirm current results.

## Figures and Tables

**Table 1:** Normal lymph nodes and lymph node metastases.

Patient	Tissue Type	Hormonal Therapy	Radiotherapy	PS	IS	TIS
1	Lymph node normal	No	No	0	0	0
	Lymph node metastasis	No	No	4	2	8
2	Lymph node normal	No	No	0	0	0
	Lymph node normal	No	No	0	0	0
	Lymph node metastasis	No	No	4	2	8
	Lymph node metastasis	No	No	4	2	8
3	Lymph node normal	No	No	0	0	0
	Lymph node metastasis	No	No	4	3	12
4	Lymph node normal	No	No	0	0	0
	Lymph node metastasis	No	No	3	1	3
5	Lymph node normal	No	No	0	0	0
	Lymph node metastasis	No	No	4	3	12
6	Lymph node normal	No	No	0	0	0
	Lymph node metastasis	No	No	3	3	9
7	Lymph node normal	No	No	0	0	0
	Lymph node metastasis	No	No	4	2	8
8	Lymph node normal	No	No	0	0	0
	Lymph node metastasis	No	No	4	3	12
9	Lymph node normal	No	No	0	0	0
	Lymph node normal	No	No	0	0	0
	Lymph node normal	No	No	0	0	0
	Lymph node metastasis	No	No	4	2	8
	Lymph node metastasis	No	No	4	2	8
	Lymph node metastasis	No	No	4	3	12
10	Lymph node normal	No	No	0	0	0
	Lymph node normal	No	No	0	0	0
	Lymph node metastasis	No	No	3	2	6
	Lymph node metastasis	No	No	3	1	3
11	Lymph node normal	No	No	0	0	0
	Lymph node metastasis	No	No	3	1	3
12	Lymph node normal	No	No	0	0	0
	Lymph node metastasis	No	No	4	3	12
13	Lymph node normal	No	No	0	0	0
	Lymph node metastasis	No	No	4	2	8
14	Lymph node normal	No	No	0	0	0
	Lymph node metastasis	No	No	3	3	9
15	Lymph node normal	Yes	Yes	0	0	0
	Lymph node normal	Yes	Yes	0	0	0
	Lymph node metastasis	Yes	Yes	4	3	12
	Lymph node metastasis	Yes	Yes	3	3	9
16	Lymph node normal	No	No	0	0	0
	Lymph node metastasis	No	No	4	2	8

PS: proportion score; IS: intensity score; TIS: total immunostaining score.

**Table 2:** Bone metastases.

Patient	Tissue Type	Hormonal Therapy	Radiotherapy	PS	IS	TIS
1	Bone metastasis	Unknown	Unknown	4	3	12
2	Bone metastasis	Unknown	Unknown	4	3	12
3	Bone metastasis	Yes	No	4	3	12
4	Bone metastasis	Unknown	Unknown	4	3	12
5	Bone metastasis	Yes	No	4	3	12
6	Bone metastasis	No	No	4	2	8
7	Bone metastasis	Unknown	Unknown	0	0	0
8	Bone metastasis	Yes	Yes	4	2	8
9	Bone metastasis	Unknown	Unknown	4	3	12
10	Bone metastasis	Yes	No	3	2	6
11	Bone metastasis	Unknown	Yes	4	3	12
12	Bone metastasis	Yes	Yes	4	3	12
13	Bone metastasis	Yes	Yes	4	3	12
14	Bone metastasis	No	No	4	3	12
15	Bone metastasis	Yes	No	3	3	9
16	Bone metastasis	Unknown	Yes	4	3	12
17	Bone metastasis	Unknown	Unknown	1	1	1
18	Bone metastasis	No	No	4	3	12
19	Bone metastasis	No	No	4	3	12
20	Bone metastasis	No	No	4	3	12

PS: proportion score; IS: intensity score; TIS: total immunostaining score.

**Table 3:** Normal bone.

Patient	Tissue Type	Hormonal Therapy	Radiotherapy	PS	IS	TIS
1	Normal bone	Unknown	Unknown	0	0	0
2	Normal bone	Unknown	Unknown	0	0	0
3	Normal bone	Unknown	Unknown	0	0	0
4	Normal bone	Unknown	Unknown	0	0	0
5	Normal bone	Unknown	Unknown	0	0	0
6	Normal bone	Unknown	Unknown	0	0	0
7	Normal bone	Unknown	Unknown	0	0	0
8	Normal bone	Unknown	Unknown	0	0	0
9	Normal bone	Unknown	Unknown	0	0	0
10	Normal bone	Unknown	Unknown	0	0	0
11	Normal bone	Unknown	Unknown	0	0	0
12	Normal bone	Unknown	Unknown	0	0	0
13	Normal bone	Unknown	Unknown	0	0	0
14	Normal bone	Unknown	Unknown	0	0	0

PS: proportion score; IS: intensity score; TIS: total immunostaining score. Epithelial cell adhesion molecule (EpCAM) expression was observed in 21 out of 21 lymph node metastases (100%) and was absent in 21 out of 21 matched normal lymph nodes (100%). Median EpCAM expression (TIS) in lymph node metastases was 8. Even after hormonal therapy and radiotherapy EpCAM expression was high in 2 lymph node metastases of a single patient (patient 15). Heterogeneous EpCAM expression was seen in 3 lymph node metastases of patient 9, in 2 lymph node metastases of patient 10, and in 2 lymph node metastases of patient 15.
